# Impact of Sulfur Deficiency and Excess on the Growth and Development of Soybean Seedlings

**DOI:** 10.3390/ijms252011253

**Published:** 2024-10-19

**Authors:** Jingwen Zhou, Huimin Zhang, Yifan Huang, Shuang Jiao, Xiangmin Zheng, Wentian Lu, Wenjing Jiang, Xi Bai

**Affiliations:** 1 College of Life Science, Northeast Agricultural University, Harbin 150030, China; zhoujingwen@neau.edu.cn (J.Z.); s220901001@neau.edu.cn (H.Z.); s230901095@neau.edu.cn (Y.H.); s230901084@neau.edu.cn (X.Z.); b240901013@neau.edu.cn (W.L.); s230902010@neau.edu.cn (W.J.); 2 Key Laboratory of Soybean Molecular Breeding, Northeast Institute of Geography and Agroecology, Chinese Academy of Sciences, Harbin 150081, China; jiaoshuang@iga.ac.cn

**Keywords:** sulfur starvation, sulfur excess, soybean, sulfate transporter genes, sulfur metabolism

## Abstract

Sulfur is a critical element for plant growth and development, serving as a component of amino acids (cysteine and methionine), iron–sulfur clusters, proteins, glutathione, coenzymes, and auxin precursors. Deficiency or low concentrations of sulfur in the soil can lead to significant growth retardation in plants. The objective of our study was to examine the effects of sulfur (S) deficiency and excess on morphological symptoms, sulfur and nitrogen (N) metabolism, as well as antioxidant activity in soybean. We found that S starvation decreased the fine root length, biomass, and activity, and the chlorophyll content was reduced, while excess sulfur promotes lateral root growth. In contrast to sulfur excess, sulfur deficiency inhibits N and S metabolism levels in both subsurface and above-ground parts, and induced the expression of some sulfur transporters (SULTRs). In this study, we created soybean hairy root lines overexpressing the SULTR gene (*GmSULTR2;1a*) to observe metabolic changes following sulfur deficiency treatment. The results showed that *GmSULTR2;1a* saved the sulfur-deficient phenotype, and the antioxidant enzyme activity was much higher than that of the wildtype in the absence of sulfur. Our study revealed the important role of sulfur element in soybean growth and development and the regulation of sulfur deficiency by *GmSULTR2;1a*.

## 1. Introduction

Sulfur (S) is a crucial element required for plant growth and development [1]; a deficiency in sulfur can restrict agricultural yield and quality in productive systems [2]. Consequently, sulfur-containing fertilizers are now widely used globally to enhance crop yield and quality. Soybeans, a crucial source of oil and protein, often have low levels of sulfur-containing amino acids, which can result in nutritional imbalances. Sulfur is a critical component of sulfur-containing amino acids, such as cysteine and methionine. Cysteine acts as a precursor for various further reduced sulfur-containing compounds, including methionine, S-adenosylmethionine, and glutathione (GSH) [3,4]. GSH performs multiple critical functions within the plant cell, acting as a redox regulator, antioxidant, and defense compound [2].

Plants exhibiting S deficiency during their vegetative growth phase typically manifest initial yellowing of young leaves and reduced growth [5]. The impact of sulfur deficiency on rice encompasses alterations in photosynthesis, metabolism, and gene expression, with a primary focus on mechanisms associated with energy generation (through photosynthesis), energy preservation (via carbohydrate metabolism), and defense against oxidative stress (employing antioxidants) [6].

Sulfur deficiency affects N:S ratios, subsequently exerting a negative influence on quality [7]. A deficiency in sulfur reduces the uptake of nitrogen (N), magnesium (Mg), and potassium (K), and vice versa. The most well-documented effect is the adverse impact of sulfur deficiency on overall nitrogen metabolism, including its uptake and assimilation, which consequently affects the growth, yield, and quality of harvested products in most cultivated species [8]. The application of sulfate has been shown to increase the total dry mass of the plant, root length, and nodule biomass, as measured by the root length of white clover [9]. High sulfur (S) fertilization enhances the levels of Rubisco, chlorophyll, and protein in the fully expanded upper leaves of *Brassica juncea* L. (mustard) and *Brassica campestris* L. [10].

Sulfate serves as the primary source of sulfur absorbed by plant roots, with its uptake predominantly facilitated by the high-affinity sulfate transporters *AtSULTR1;1* and *AtSULTR1;2*, and, to a lesser extent, by the low-affinity transporter *AtSULTR2;1* [11,12]. Additionally, *AtSULTR2;1* plays a crucial role in the translocation of sulfate from roots to shoots for assimilation and storage in vacuoles, in conjunction with *AtSULTR3.5*, *AtSULTR4.1*, and *AtSULTR4.2* [13]. Upon being transported into root cells, sulfate is either conveyed into plastids via *SULTR3*, where it undergoes assimilation into organic sulfur compounds, or sequestered into vacuoles for storage [14]. Empirical studies have demonstrated that *GmSultr1;2b* in soybeans facilitates sulfur uptake and enhances plant tolerance to sulfur deficiency stress [15]. In curly kale and poplar, the sulfur status of plants modulates the activity and expression of sulfate transporters and APS reductase. Transferring seedlings to sulfate-deprived conditions resulted in a threefold increase in the root’s sulfate uptake capacity and elevated the transcript levels of Group 1 and Group 4 sulfate transporters in both root and shoot tissues [16,17].

In conclusion, it is imperative to investigate the morphological and physiological adaptations of soybean to sulfur deprivation. Sulfur deficiency and surplus represent distinct mechanisms of sulfur provision, each exerting differential impacts on sulfur uptake and metabolism in plants. The following hypotheses were posited in this study: (i) sulfur deprivation and excess induce morphological and physiological changes in the roots, stems, and leaves of soybean plants; (ii) sulfur deficiency and excess conditions affect soybean sulfur and nitrogen metabolism, thereby modulating growth. To evaluate these two hypotheses, we examined the morphological symptoms, as well as the metabolic and transcriptional changes in soybean induced by sulfur deficiency and excess. Soybean plants were treated with S deficiency and S excess for a duration of four weeks. The results indicated that sulfur treatment significantly influenced morphological symptoms, sulfur and nitrogen metabolism, and oxidative stress processes. Additionally, the soybean sulfur transporter gene *GmSULTR2;1a* was identified following sulfur deficiency treatment and subsequent sulfur application, with its expression being upregulated. We created *GmSULTR2;1a*-overexpressed hairy root material and found that it could improve the damage to soybean growth caused by sulfur deficiency. The overexpression of this gene in hairy root material was found to mitigate the adverse effects of sulfur deficiency on soybean growth.

## 2. Results

### 2.1. Sulfur Influences Soybean Morphology, Photosynthesis, and Growth

Soybean seeds were germinated for 5 days under S deficiency (−S, 0 mM SO_4_^2−^), S control (CK, 2 mM SO_4_^2−^), or S excess (+S, 4 mM SO_4_^2−^) conditions. A comparison of radicle lengths under the different treatments revealed that, compared to the control, sulfur application did not result in significant changes. However, sulfur deficiency limited the growth of the radicle (Figure S1).

**Figure 1:**
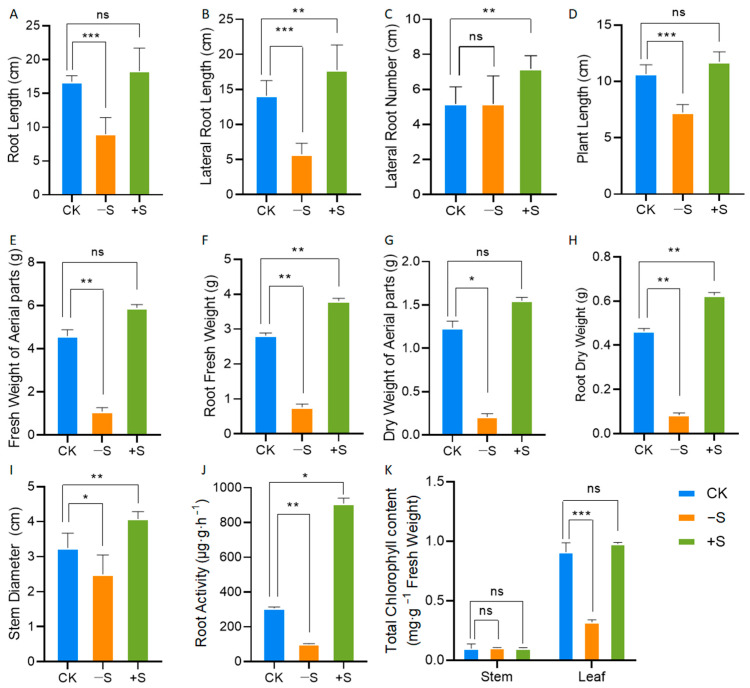
Morphological, physiological, and growth responses to CK, −S, or +S conditions. Root length (**A**), lateral root length (**B**), lateral root number (**C**), plant length (**D**), fresh weight of aerial parts (**E**), root fresh weight (**F**), dry weight of aerial parts (**G**), root dry weight (**H**), stem diameter (**I**), root activity (**J**), and total chlorophyll content (**K**) in soybean. Stars indicate significant difference (Student’s *t*-test, * *p* < 0.05, ** *p* < 0.01 and *** *p* < 0.001) compared to control. And “ns” indicate “not significant”.

Following a four-week exposure to −S, CK, or +S conditions in standard hydroponic medium, notable alterations were observed in the morphology, photosynthesis, and growth of soybean saplings (Figure 1 and Figure S2). Specifically, sulfur deprivation significantly inhibited the length, biomass, and activity of soybean roots (Figure 1A,B,F,H,J), whereas they were stimulated by S excess (Figure 1B,C,F,H,J). The plant length, stem diameter, biomass above ground, and the leaf chlorophyll of soybean were lower under S starvation (Figure 1D,E,G,I,K); however, the application of sulfur did not alter the height and biomass of the above-ground parts (Figure 1D,E,G). The chlorophyll of the stem was not affected by both S starvation and S excess, whereas it decreased in response to S starvation in the leaves (Figure 1K).

### 2.2. Sulfur Significantly Impacts N and S Metabolism

**Figure 2:**
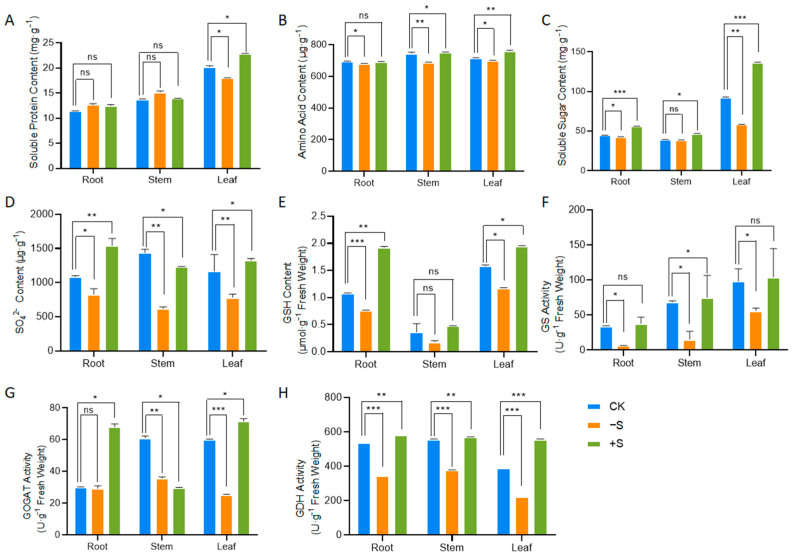
Changes in N and S metabolism in soybean seedlings under sulfur deprivation and excess. Soluble protein content (**A**), amino acid content (**B**), soluble sugars content (**C**), sulfate (SO_4_^2−^) (**D**), glutathione (GSH) content (**E**), GS activity (**F**), GOGAT activity (**G**), and GDH activity (**H**) in soybean under CK, −S, and +S. Stars indicate significant difference (Student’s *t*-test, * *p* < 0.05, ** *p* < 0.01 and *** *p* < 0.001) compared to control. And “ns” indicate “not significant”.

Following sulfur application, there was a significant increase in the content of soluble proteins in the leaves, whereas under sulfur-deficient conditions, the content of soluble proteins in the leaves decreased (Figure 2A); concurrently, the total amino acid content declined in roots, stems, and leaves (Figure 2B). In terms of soluble sugars, sulfur application led to an increase in their content in both above- and below-ground parts, while sulfur deficiency had the opposite effect (Figure 2C). We observed that the levels of sulfate (SO_4_^2−^) and glutathione (GSH) significantly decreased under sulfur-deficient conditions (Figure 2D,E), with glutathione playing a crucial role in antioxidant defense [18]. These alterations may potentially impact the growth status of soybean plants. In order to investigate the impact of sulfur levels on nitrogen metabolism, we measured the enzyme activities of glutamine synthetase (GS), glutamate synthase (GOGAT), and glutamate dehydrogenase (GDH). We found that their activities were constrained under sulfur-deficient conditions, which inhibited nitrogen metabolism, while sulfur application promoted nitrogen metabolism (Figure 2F–H).

**Figure 3:**
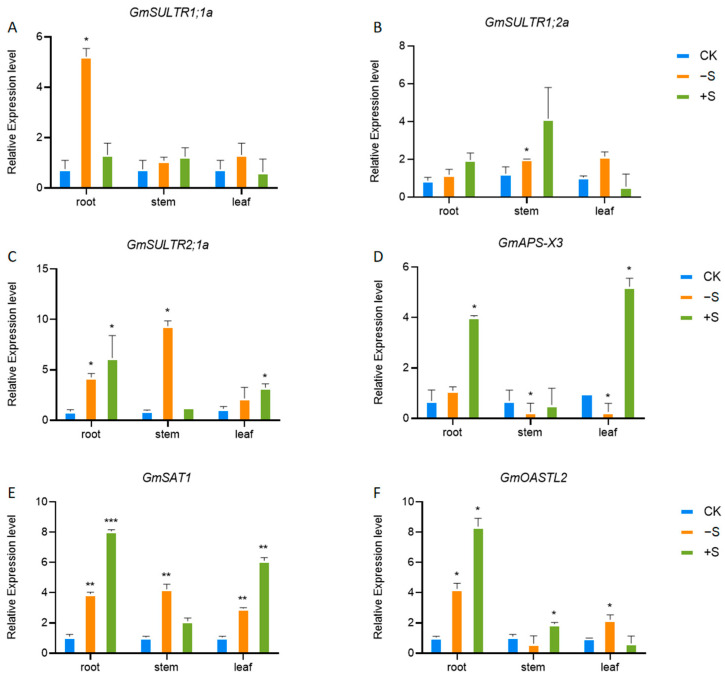
Expression of key genes in sulfur assimilation and cysteine synthesis pathway under sulfur-excess (+S) and sulfur-deficiency (−S) conditions. Relative expression level of *GmSULTR1;1a* (**A**), *GmSULTR1;2a* (**B**), *GmSULTR2;1a* (**C**), *GmAPS-X3* (**D**), *GmSAT1* (**E**), and *GmOASTL2* (**F**) in soybean under CK, −S, and +S. Stars indicate significant difference (Student’s *t*-test, * *p* < 0.05, ** *p* < 0.01 and *** *p* < 0.001) compared to control.

The expression of key biosynthetic genes in the sulfur assimilation pathway is also affected by sulfur content. Sulfate transporters are key proteins in plants responsible for the transport and absorption of sulfate. *GmSULTR1;1a* exhibits enhanced responsiveness to sulfur (S) starvation in roots, while *GmSULTR1;2a* is upregulated in stems under S-deprived conditions. Neither gene is expressed when plants are cultivated in environments with high external sulfate concentrations (Figure 3A,B). Conversely, *GmSULTR2;1a* expression is markedly induced by S starvation in both roots and stems (Figure 3C). ATP sulfurylase (*GmAPS-X3*) is the first step in the sulfur assimilation pathway, which converts inorganic sulfates into organic sulfides APS, providing essential active sulfates for subsequent sulfur metabolic reactions [19]. However, in the absence of sulfur, APS was inhibited in the aboveground parts of plants, and on the contrary, it promoted the expression of APS during sulfur application (Figure 3D), similar to *Arabidopsis* and *Brassica napus* [20,21]. Sulfur is also crucial to cysteine synthesis. We detected the expression levels of serine acetyltransferase (*GmSAT1*) and cysteine synthase (*GmOASTL2*), key enzyme genes in the process of cysteine synthesis, under the condition of sulfur deficiency and sulfur application, and found that both underground and above-ground expressions of both can be induced under both conditions (Figure 3E,F).

### 2.3. Sulfur Starvation and Excess Affect the Antioxidant Levels 

**Figure 4:**
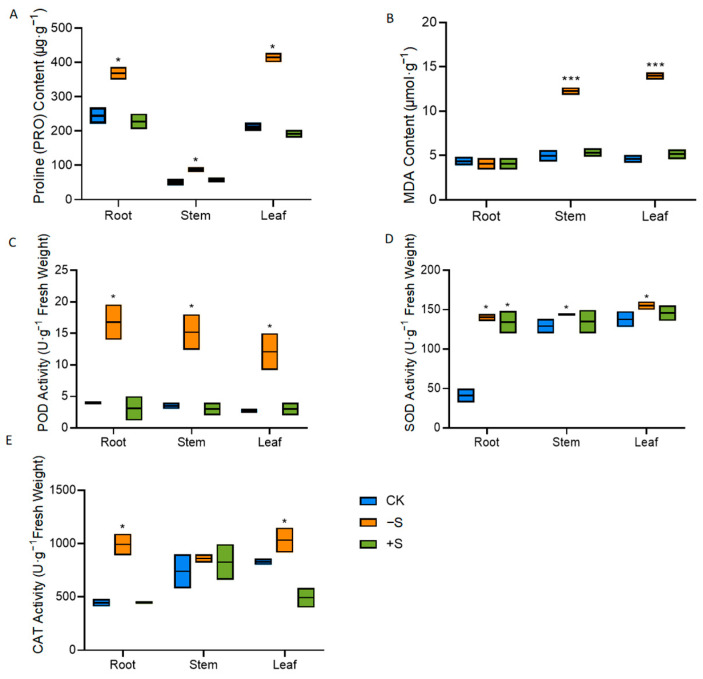
The effect of sulfur deficiency (−S) and excess (+S) on proline content (**A**), malondialdehyde content (**B**), activity of peroxidase (**C**), superoxide dismutase (**D**), and catalase (**E**) in soybean. Stars indicate significant difference (Student’s *t*-test, * *p* < 0.05 and *** *p* < 0.001) compared to control.

Proline (Pro) serves as a biomarker for plant stress and is crucial for plant adaptation under stress conditions [22]. As illustrated in Figure 4A, proline levels in various plant tissues were significantly elevated in response to sulfur deficiency, while proline levels remained unchanged when sulfur was adequately supplied. Malondialdehyde (MDA) is a key indicator of membrane lipid peroxidation [22]. The data demonstrate that MDA levels in the above-ground parts of the plant increased under sulfur-deficient conditions. However, no significant variation in MDA content was observed when sulfur was supplied in excess (Figure 4B).

In addition, we examined the activity of antioxidant enzymes, including superoxide dismutase (SOD), peroxidase (POD), and catalase (CAT). Under the condition of sulfur deficiency, the activity of antioxidant enzymes increased. The activity of antioxidant enzymes was similar to that of the control group compared with the sulfur-treated state (Figure 4C–E). These results suggest that sulfur deficiency can cause oxidative stress and promote enhanced antioxidant enzyme activity.

### 2.4. Correlation Analysis Between S and N Metabolism on Soybean Responses to Sulfur Deficiency or Excess Sulfur

**Figure 5:**
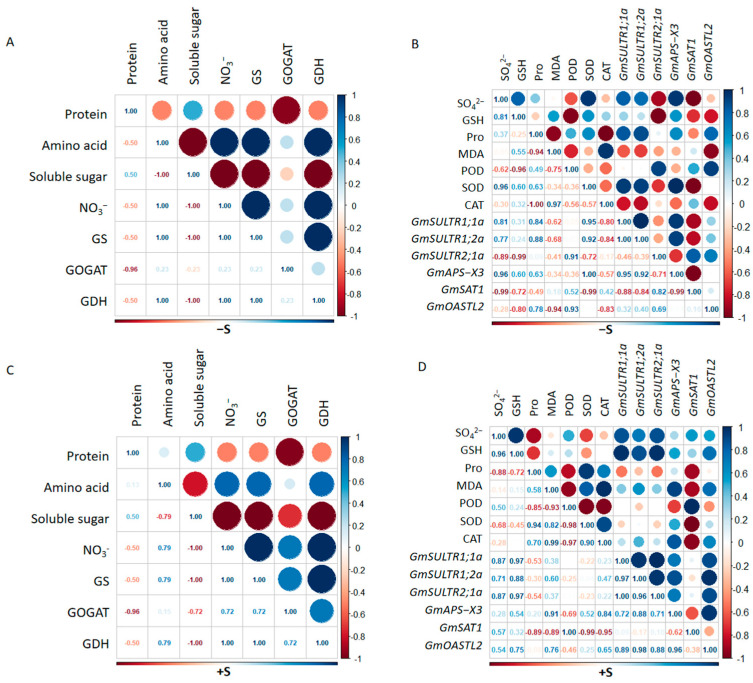
Correlation analysis of multiple variables. Correlation matrix of sulfur- and nitrogen-related trait data from soybean grown in −S (**A**,**B**) and +S (**C**,**D**). The results are presented as a heat map with the Pearson correlation score from –1 to 1.

In order to comprehensively analyze the effects of sulfur deficiency and excess on soybeans, correlation analysis was performed on plant S and N metabolism and enzymatic activities. In terms of nitrogen metabolism, NO_3_^−^ and amino acid contents showed strong positive correlation with N metabolism-related enzymes in different sulfur states, but the correlation was stronger when S was absent (Figure 5A,C). In the sulfur deficiency treatment, SO_4_^2−^ content was highly correlated with glutathione (GSH) content and SULTR1, and negatively correlated with *GmSULTR2;1a* (Figure 5B). On the contrary, in the sulfur application treatment, SO_4_^2−^ and GSH were highly correlated with genes in the sulfur assimilation pathway, and the correlation between sulfur metabolism genes and metabolites was higher than that in the sulfur deficiency treatment (Figure 5D). However, there was no strong correlation between GSH and antioxidant-related indexes under different sulfur treatments. Through correlation analysis and combined with the above data, we found that different S treatments had different effects on S and N metabolism.

### 2.5. Overexpression of GmSULTR2;1a Alleviated Soybean Phenotype Under Sulfur Deficiency

**Figure 6:**
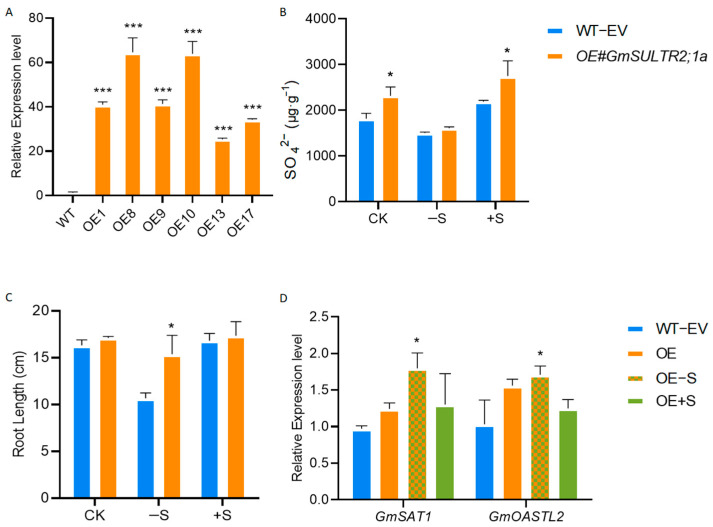
Analysis of sulfur deficiency and excess in *GmSULTR2;1a*−overexpressed soybean hairy roots. (**A**) qRT-PCR analysis of *GmSULTR2;1a* transcript levels in transgenic hairy roots. SO_4_^2−^ content (**B**), root length (**C**), and *GmSAT1 and GmOASTL2* transcript levels in *GmSULTR2;1a*−overexpressed soybean hairy root material. (**D**) Relative expression level of *GmSAT1* and *GmOASTL2* in *GmSULTR2;1a*-overexpressed soybean hairy roots under CK, −S, and +S. WT−EV was obtained by infection with K599 with 35S−EGFP empty vector. Stars indicate significant difference (Student’s *t*-test, * *p* < 0.05 and *** *p* < 0.001) compared to control.

To elucidate the role of *GmSULTR2;1a* in sulfur transport mechanisms, we generated transgenic soybean hairy root lines overexpressing *GmSULTR2;1a* (*OE*#*GmSULTR2;1a*). The control group (WT-EV) was infected with DN50 using the pCAMBIA1300-35S-EGFP empty vector. The successful integration of *GmSULTR2;1a* into the soybean hairy roots was confirmed through quantitative reverse transcription PCR (qRT-PCR). The qRT-PCR analysis revealed that *GmSULTR2;1a* was transcriptionally active in the transgenic soybean hairy roots, with expression levels ranging from 25- to 69-fold (Figure 6A). Under control and sulfur-excess conditions, the SO_4_^2−^ content was elevated compared to the WT-EV (Figure 6B); under sulfur-deficient conditions, the root length of *OE#GmSULTR2;1a* surpasses that of the control group (Figure 6C). *GmSULTR2;1a* can induce upregulated expression of important genes *GmSAT1* and *GmOASTL2* in the sulfur assimilation pathway when sulfur is deficient (Figure 6D). These findings indicate that the *GmSULTR2;1a* gene enhances the growth performance of soybeans under sulfur-deficient conditions.

### 2.6. Overexpression of GmSULTR2;1a Alleviated Oxidative Stress Induced by Sulfur Deficiency 

**Figure 7:**
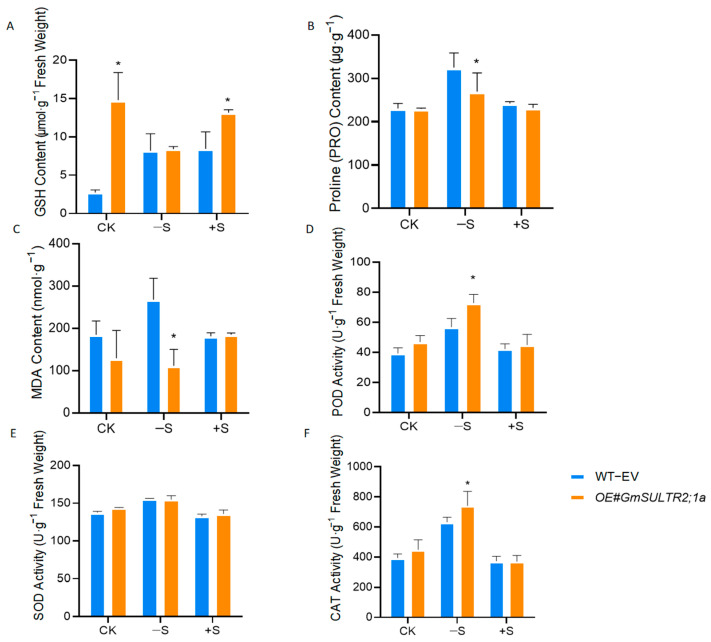
The effect of sulfur deficiency (−S) and excess (+S) on GSH content (**A**), proline content (**B**), malondialdehyde content (**C**), activity of peroxidase (**D**), superoxide dismutase (**E**), and catalase (**F**) in WT−EV and *35S::GmSULTR2;1a*. Stars indicate significant difference (Student’s *t*-test, * *p* < 0.05) compared to control.

Glutathione (GSH) is a crucial sulfur-containing metabolite, and the thiol group of the cysteine residue in GSH imparts its antioxidant properties [18]. To investigate whether *GmSULTR2;1a* mediates oxidative stress induced by sulfur deficiency, we analyzed the GSH content in *OE#GmSULTR2;1a*. Under normal and sulfur-excess conditions, the GSH levels in the transgenic hairy roots were significantly higher than those in the control group. However, no significant change in GSH content was observed under sulfur-deficient conditions (Figure 7A).

We additionally quantified the levels of MDA and Pro, alongside the activities of antioxidant enzymes. The results indicated that sulfur deficiency led to an elevation in both MDA and Pro contents. However, this increase was comparatively minor in the *OE#GmSULTR2;1a* transgenic lines, suggesting that the overexpression of *GmSULTR2;1a* confers a protective effect on the cell membrane (Figure 7B,C). Furthermore, we assessed the activity of antioxidant enzymes, specifically superoxide dismutase (SOD), peroxidase (POD), and catalase (CAT) (Figure 7D–F). A significant increase in the activity of these antioxidant enzymes was observed under sulfur deficiency, with the exception of SOD. Compared to the wildtype, the *OE#GmSULTR2;1a* lines exhibited a more pronounced increase in antioxidant enzyme activity (Figure 7D–F). The application of sulfur caused no significant change in the antioxidant system (Figure 7B–F). These findings suggest that the overexpression of *GmSULTR2;1a* enhances antioxidant enzyme activity, thereby mitigating oxidative stress induced by sulfur deficiency.

## 3. Discussion

In this investigation, soybean seedlings were cultivated in pots using hydroponic solutions with varying sulfate concentrations to compare the effects of sulfate levels under normal sulfur, sulfur-deficient (−S), and sulfur-excess (+S) conditions. S deficiency symptoms first appear in young plant parts due to poor S mobility. While symptoms vary by plant type, they generally include reduced height and chlorosis, particularly in new leaves [23]. The effects of sulfur deficiency on plant root morphology have been extensively investigated. In *Arabidopsis*, lateral root development was markedly inhibited, as demonstrated by a significant reduction in both the number of lateral roots and the density of lateral root primordia and lateral roots [23]. Moreover, the application of sulfur has been shown to increase root length and root surface area in alfalfa grown in soils with low sulfur availability [24]. Similarly, S starvation significantly inhibited soybean root length, biomass, and activity, while S excess stimulated them (Figure 1A–C,F,H,J). S starvation also reduced plant length, stem diameter, and above-ground biomass (Figure 1D,E,G,I).

Numerous studies have examined plant physiological responses to sulfur (S) nutrition [25]. As S is crucial for proteins, chloroplasts, and essential enzymes, its deficiency reduces S content and S-containing amino acids, thereby lowering metabolic activity [26]. In the advanced stages of oilseed rape (*Brassica juncea* L. and *Brassica campestris* L.) development, sulfur deficiency impedes growth and diminishes leaf count. Chlorosis may manifest in the young leaves, accompanied by a reduction in photosynthetic activity. Conversely, elevated sulfur fertilization enhances the levels of Rubisco, chlorophyll, and proteins in the upper leaves of these species [27]. In our study, sulfur application significantly boosted soluble protein content in leaves, while sulfur deficiency reduced it (Figure 2A). Total amino acids decreased in roots, stems, and leaves (Figure 2B). S metabolism is intricately linked with C and N assimilation [28]. S nutrition is closely tied to N, being essential for protein synthesis (K). Hence, sulfate deficiency curbs nitrate uptake and reduction, and conversely, N deficiency decreases sulfate uptake and reduction rates [28].To study the effect of sulfur on nitrogen metabolism, we measured the activities of enzymes GS, GOGAT, and GDH. We discovered that sulfur deficiency reduced their activities and inhibited nitrogen metabolism, whereas adding sulfur enhanced it (Figure 2F–H). Through correlation analysis, it was also found that N metabolism was closely related to the activities of enzymes GS, GOGAT, and GDH (Figure 5A,C). This S deficiency impairs key enzyme synthesis in carbon metabolism, slows photosynthesis, and increases reactive oxygen species accumulation in plants [29,30]. In this study, S starvation also reduced chlorophyll content of leaves (Figure 1K). Soluble sugars increased with sulfur application in all plant parts, but decreased under sulfur deficiency (Figure 2C). We found that the level of sulfur had a significant effect on the related indices of N metabolism.

On the other hand, sulfur concentration also affects sulfur metabolism, with GSH as the primary form of reduced S storage and transport [31]. GSH acts as a systemic signal for sulfate assimilation regulation in demand-driven processes in *Brassica napus* and *Arabidopsis* [32,33]. Sulfur deprivation treatment induced symptoms indicative of sulfur deficiency [19,24,34,35], where S-deprived plants were characterized by decreased sulfate (Figure 2D) and reduced GSH (Figure 2E). Sulfate deficiency triggers enhanced sulfate uptake and activation of the sulfate transporters *ATPS* and *APR* in plant cells, along with upregulation of chloroplastic *SAT* and cytoplasmic *OASTL* mRNA levels due to sulfur scarcity [36,37]. In our study, the expression of key synthetic genes (*GmAPS*, *GmSAT1*, and *GmOASTL2*) in the sulfur assimilation pathway is influenced by sulfur levels (Figure 3). Sulfate transporters are crucial for sulfate uptake in plants. *GmSULTR2;1a* is significantly induced by sulfur starvation in roots and stems (Figure 3C). *AtSULTR2;1* expression is significantly increased in roots under sulfur starvation, but not in leaves. Suppressing *SULTR2;1* with antisense RNA reduces sulfate content in seeds [38]. We generated hairy root materials of soybean with *GmSULTR2;1a* and found that its sulfate content was significantly higher than those of the control (Figure 6B). *GmSULTR2;1a* can induce the expression of *GmSAT1* and *GmOASTL2*, thus promoting the degree of sulfur metabolism (Figure 6D). Plants will produce reactive oxygen species (ROS) under stress, and the concentration of malondialdehyde (MDA) and the content of proline (Pro) reflect the degree of oxidative stress. Plants have enzymatic mechanisms to detoxify ROS. Peroxidase (POD), superoxide dismutase (SOD), and hydrooxidase (CAT) can be detoxified [30,36]. GSH, a key regulator of cellular redox potential, significantly contributes to the intricate gene network that shields cells from oxidative stress [39]. A decrease in GSH content in response to sulfur deficiency has been documented across various plant species, including rapeseed, barley, and *Arabidopsis* [40,41,42]. Our study suggests that the antioxidant capacity of *GmSULTR2;1a* was also higher than that of the wildtype with high GSH and antioxidant enzyme activity under sulfur deficiency (Figure 7A–E). Therefore, our study provides a valuable reference to better understand the effects of sulfur on the growth of soybean seedlings and the salvage role of sulfur transporters *GmSULTR2;1a* in the condition of sulfur deficiency.

## 4. Materials and Methods

### 4.1. Plant Materials and Culture Conditions

Soybean seeds of the “Dongnong50” cultivar (Genetic Engineering Laboratory of the College of Life Sciences, Northeastern Agricultural University, Harbin, China) were germinated in plastic containers filled with double-distilled water (ddH_2_O). After one week, the seedlings were transferred to a standard hydroponic medium (Coolaber, Beijing, China) containing 2 mM MgSO_4_. The sulfur-deprived plants were cultivated in nutrient solutions analogous to the control group, with the exception that sulfate (SO_4_^2−^) salts were substituted with equivalent concentrations of chloride salts, thereby rendering the solutions sulfur-deficient. Conversely, sulfur excess was induced by supplementing the standard hydroponic medium with additional 2 mM MgSO_4_. These sulfur treatments were maintained for a duration of four weeks until the end of the experiment.

### 4.2. Analysis of Morphological Characteristics

Plant height and stem diameter were measured at the V4 (fourth trifoliate stage). The roots of each plant were meticulously cleaned and dried using paper towels to eliminate surface moisture. Subsequently, root length, fresh weight, and dry weight were recorded. The fresh and dry weights of the above-ground parts of these plants were also measured. Unless otherwise specified, samples of roots, stems, and leaves formed during the S-treatment were utilized for molecular and biochemical analyses. For each tissue type, equal weights of finely powdered samples, derived from three plants subjected to identical treatments, were combined to create a homogenized sample. Root activity was subsequently assessed using a kit from Solarbio (Beijing Solarbio Science & Technology Co., Ltd., BC5270, Beijing, China).

### 4.3. Determination of Amino Acids, Soluble Proteins, Chlorophyll, and Sugars

Free amino acids were analyzed according to the method of Boxbio Amino Acid (AA) Content Assay Kit (AKAM001M). The soluble protein contents were analyzed based on the Bradford method [43]. The concentrations of chlorophyll in the leaves were determined spectrophotometrically [44]. The concentrations of soluble sugars in roots, stems, and leaves were determined by the anthrone method with minor modifications [45].

### 4.4. Analyses of Metabolites and Enzyme Activity Assays

The concentration of SO_4_^2−^ was quantified following the turbidimetric method [46]. The levels of GSH were assessed using the Solarbio company kit (AKPR008M) [47]. The GS (EC 6.3.1.2), GOGAT (EC 1.4.7.1), and GDH (EC 1.4.1.2) activities levels in roots, stems, and leaves were determined according to the method with minor modifications [48].

### 4.5. Total RNA Extraction and qRT-PCR Analysis

Gene transcription changes related to sulfur uptake and assimilation were analyzed using reverse-transcription quantitative PCR (qPCR), as described in the methods of Ref. [49]. Briefly, total RNA was isolated from 100 mg of frozen root and stem powder and 50 mg of leaf powder using EasyPure^®^ (Beijing, China) Plant RNA Kit (ER301-01). A mass of 1 μg of total RNA was then used for first-strand cDNA synthesis with TransScript^®^ (Beijing, China) One-Step SuperMix (AT411-02) in a 20 μL reaction. qRT-PCR was conducted on a LightCycler R 96 with TransStart^®^ (Beijing, China) Top Green qPCR SuperMix (AQ131-01), using *GmTUA5* as the reference gene. Primer details are in Table S1. The experiment included three biological and technical replicates, and data were analyzed using the $2^{-∆∆ct}$ method [38].

### 4.6. Preparation of Transgenic Soybean Hairy Roots

The coding sequences of *GmSULTR2;1a* were cloned into the pCAMBIA1300-35S-EGFP vector and subsequently introduced into Agrobacterium rhizogenes strain K599. The soybean cultivar DongNong50 was employed for transformation, which was conducted under a photoperiod of 16 h light and 8 h dark at a temperature of 25 °C within a humidity-controlled chamber. The control group (EV) was infected with DN50 using the pCAMBIA1300-35S-EGFP empty vector. This procedure resulted in the development of transformed hairy roots [50]. Verification of the hairy roots was performed using quantitative reverse transcription PCR (qRT-PCR), leading to the identification of positive transgenic soybean hairy roots.

### 4.7. Antioxidant Enzyme Activity Assay

Peroxidase (POD) activity was measured at 470 nm using an assay kit from Beijing Boxbio Science & Technology Co., Ltd. (Beijing, China) (AKAO005C). Superoxide dismutase (SOD) activity was measured at 560 nm using their kit (AKAO001M) via the hydroxylamine method. Catalase (CAT) activity was measured at 240 nm using their kit (AKAO003-1M). PRO content was measured at 520 nm using an assay kit (Beijing Boxbio Science & Technology Co., Ltd., AKAM003C, Beijing, China). MDA activity was measured at 532 nm, 450 nm, and 600 nm using a colorimetric assay kit (Beijing Boxbio Science & Technology Co., Ltd., AKFA013C, Beijing, China). Calculation formulas for enzyme activities were provided in the instruction manuals.

### 4.8. Statistical Analysis

Statistical analyses were performed using SPSS version 23.0 software. Experiments were conducted in triplicate, and the results are presented as mean ± standard deviation (SD). The Student’s *t*-test was employed to determine *p* values, with statistical significance defined as *p* < 0.05. Graphical representations were generated using GraphPad Prism version 8.0.1. The Pearson correlation analysis was performed using the ‘Hmisc’ and ‘corrplot’ packages in R: https://www.R-project.org (accessed on 15 July 2024).

## 5. Conclusions

Our results show that S element levels significantly affect plant growth status, including phenotype, metabolism, and gene expression. S-starvation reduced fine root length, biomass, and activity, chlorophyll content decreased, and excessive sulfur promoted lateral root growth. Compared with sulfur excess, sulfur deficiency inhibited N and S metabolism levels in both subsurface and above-ground parts, and induced the expression of some sulfur assimilation pathway genes. To investigate the role of *GmSULTR2;1a* in soybean seedlings under sulfur-deficient conditions, we cultivated transgenic soybean hairy roots expressing *GmSULTR2;1a*. The results indicate that *GmSULTR2;1a* enhances sulfur metabolism and antioxidant levels, thereby mitigating the detrimental effects of sulfur deficiency on soybean growth.

## Data Availability

Data are contained within the article and Supplementary Materials.

## Supplementary Materials

The following supporting information can be downloaded at: https://www.mdpi.com/article/10.3390/ijms252011253/s1.
