# Supplementary material for: Impact of Sulfur Deficiency and Excess on the Growth and Development of Soybean Seedlings
Source: Int J Mol Sci. 2024 Oct 19;25(20):11253. doi: 10.3390/ijms252011253 (PMC11508489; doi:10.3390/ijms252011253)
Supplement: Supplementary file 1 [file ijms-25-11253-s001.zip › Supplementary Materials.pdf]

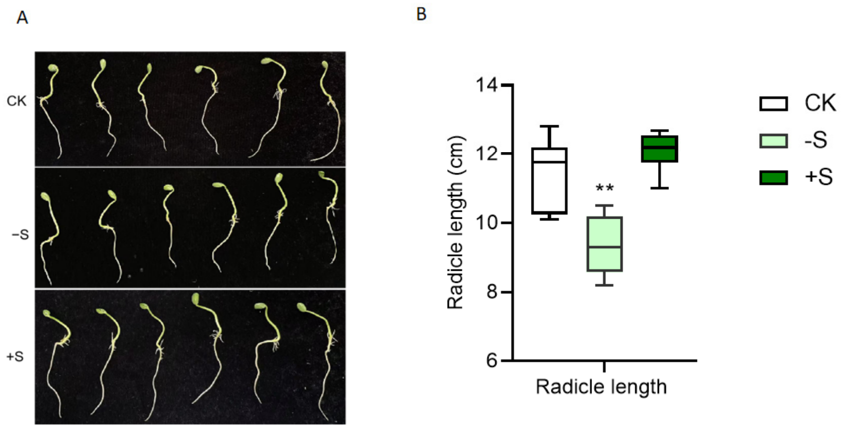

Figure S1 Radicle length of soybean under S control (CK, 2 mM SO_4^{2-}), S deficiency (−S, 0 mM SO_4^{2-}) or S excess (+S, 4 mM SO_4^{2-}) conditions.

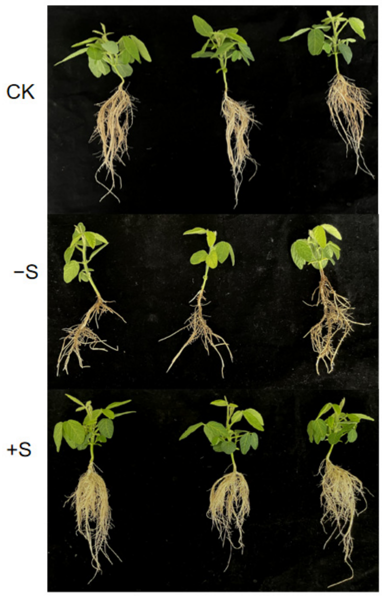

Figure S2 Morphological, growth characteristics of soybean under S control (CK, 2 mM SO_4^{2-}), S deficiency (−S, 0 mM SO_4^{2-}) or S excess (+S, 4 mM SO_4^{2-}) conditions.

Table S1 Sequences of primers used in this study

Gene	Primer
GmTUA5-F	GAGCAAAGAGATCACTGCACTTG
GmTUA5-R	CTCATATTCGGTCTTGGCAATCC
GmSULTR1;1a-F	AAGTGGCAATTCCTCCGAGAC
GmSULTR1;1a-R	CGAGCCTGATCTTCCTTGATC
GmSULTR1;2a-F	TTCTGTGGTAGACACTTTCTTCCC
GmSULTR1;2a-R	GGTGAGTCCAGAAATGAAATCACCT
GmSULTR2;1a-F	GCTGCTTTTGGACTCTTCAGGT
GmSULTR2;1a-R	CAAACGGATTTCATGACAGAGA
GmOASTL2-F	TAATGAGTCTCGAGAGGAGAATTGT
GmOASTL2-R	ATTGATGAGACATAAAAGCATCACA
GmSAT1-F	CAACAACAATTCATCCTCACACT
GmSAT1-R	GTCCACACATCGACTTCAACCTC
GmAPS-X3-F	GCCTAATGGCATCAATCAATCTACT
GmAPS-X3-R	ACGCAGCCCTTTTGCTGAAGTAGCT
